# Manual mid-stromal dissection as a low risk procedure to stabilize mild to moderate progressive keratoconus

**DOI:** 10.1186/s40662-018-0121-2

**Published:** 2018-10-11

**Authors:** Rénuka S Birbal, Korine van Dijk, Jack S Parker, Henny Otten, Maha Belmoukadim, Lisanne Ham, Lamis Baydoun, Isabel Dapena, Gerrit R J Melles

**Affiliations:** 1 Netherlands Institute for Innovative Ocular Surgery (NIIOS) Rotterdam, Laan op Zuid 88, 3071AA Rotterdam, The Netherlands; 2 Melles Cornea Clinic Rotterdam, Rotterdam, The Netherlands; 3 Amnitrans EyeBank Rotterdam, Rotterdam, The Netherlands; 4 NIIOS-USA, San Diego, USA; 5 Parker Cornea, Birmingham, AL USA; 6 Visser Contact Lens Practice Nijmegen/Rotterdam, Rotterdam, The Netherlands

**Keywords:** Keratoconus, Manual cornea dissection, Progressive ectasia, Surgical technique

## Abstract

**Background:**

To evaluate the efficacy of manual mid-stromal dissection in stabilizing progressive keratoconus.

**Methods:**

Surgeries were performed in 16 eyes of 14 patients with progressive keratoconus. All eyes were examined before and at 1 day, 1 week, 1, 3, 6 and 12 months after surgery, and every 6 months thereafter. Pentacam (simK, Kmax and pachymetry), best corrected visual acuity (BCVA) and subjective refraction were recorded up to the latest follow-up visit (mean follow-up time 6.6 ± 2.4 years).

**Results:**

All surgeries were uneventful, and no postoperative complications occurred. Keratometry values (*n* = 15) stabilized in 6/11 eyes (55%) with a preoperative Kmax < 60.0 diopter (D), while all eyes > 60 D showed continued progression. In 11/15 eyes (73%) pachymetry was unchanged. BCVA with spectacles remained stable in 7/12 eyes (58%) and improved ≥2 Snellen lines in 5/12 eyes (42%). BCVA with a contact lens remained stable in 4/9 eyes (44%), improved ≥2 Snellen lines in 3/9 eyes (33%) and deteriorated in 2/9 eyes (22%).

**Conclusions:**

Manual mid-stromal dissection was effective in 50% of keratoconic corneas with Kmax values < 60 D and may be considered in cases ineligible for other interventions such as UV-crosslinking, stromal ring implantation or Bowman layer transplantation. An advantage of the procedure may be that the tissue is unaltered and that no synthetic or biological implant is required.

## Background

Until a decade ago, keratoconus (KC) has been treated with contact lens fitting until disease progression required penetrating keratoplasty (PK) or deep anterior lamellar keratoplasty (DALK) [1]. In 2003, Wollensak et al. introduced ultraviolet-A-induced collagen crosslinking (UV-CXL) as a concept to stabilize corneal ectasia by strengthening the stromal collagenous corneal matrix [2]. Its use may be limited to keratoconic corneas that measure at least 400 μm in thickness [2]. Alternatively, intrastromal corneal ring segments (ICRS) have been described to modify the corneal contour [1]. All these procedures share the disadvantage of significantly altering the corneal anatomy which may bear the risk of potential complications in the long term [3, 4].

To offer patients a low risk alternative to halt or slow down disease progression, we introduced a different approach: ‘manual mid-stromal dissection’. We hypothesized that stabilization of corneal ectasia in eyes with keratoconus may be obtained through a wound healing effect within the stroma following manual dissection.

The aim of this study was to evaluate the efficacy of the procedure in stabilizing keratoconic corneas as well as to substantiate a significantly lower incidence of complications.

## Methods

### Patient data

A total of 16 eyes of 14 patients (6 female), with a mean age of 33.8 ± 12.1 years (range, 19–72 years), underwent manual mid-stromal dissection (Table 1) and had a mean follow-up of 6.6 ± 2.4 years (range, 1.6–9.4 years). All treated eyes had documented evidence of keratoconus progression in the year prior to surgery (defined as ≥1.0 Diopters (D) change in maximum keratometry [Kmax] values (measured by Scheimpflug-based corneal tomography [5])) with or without a history of subjective decline in visual acuity and were included in this analysis. Eyes with concomitant ocular disease not related to keratoconus and eyes with previous episodes of hydrops were excluded from treatment. All patients signed an institutional review board-approved informed consent form prior to surgery. The study was conducted according to the tenets of the Declaration of Helsinki [6].

**Table 1:** Demographics and preoperative baseline characteristics

Case no.	Age (years)/Gender	Eye	Keratoconus grade^c^	Remarks
1	38/ F	OD	I-II	
2^a^	31/ M	OS	III-IV	
3^a^	31/ M	OD	III-IV	
4	27/ M	OD	II	
5	36/ F	OS	IV	Chronic allergic conjunctivitis
6^b^	23/ M	OD	III	
7	72/ F	OS	II-III	Preexisting central corneal scarring, Diabetes mellitus type II
8	34/ M	OS	III-IV	
9	34/ M	OS	I	
10	34/ M	OS	II-III	Preexisting central corneal scarring
11	45/ M	OD	II-III	
12	26/ F	OS	I-II	
13^b^	24/ M	OS	II-III	
14	28/ F	OS	IV	Chronic allergic conjunctivitis
15	37/ F	OD	III-IV	Preexisting central corneal scarring
16	19/ M	OD	III	
Average	33.8			
SD	12.1			

*F* = female; *M* = male; *OD* = right; *OS* = left

^a,b^Note that two patients (cases no. 2, 3, 6, and 13) underwent bilateral manual crosslinking

^c^Keratoconus grading according to Pentacam Topographic Keratoconus classification [10]

### Surgical technique

**Fig. 1:**
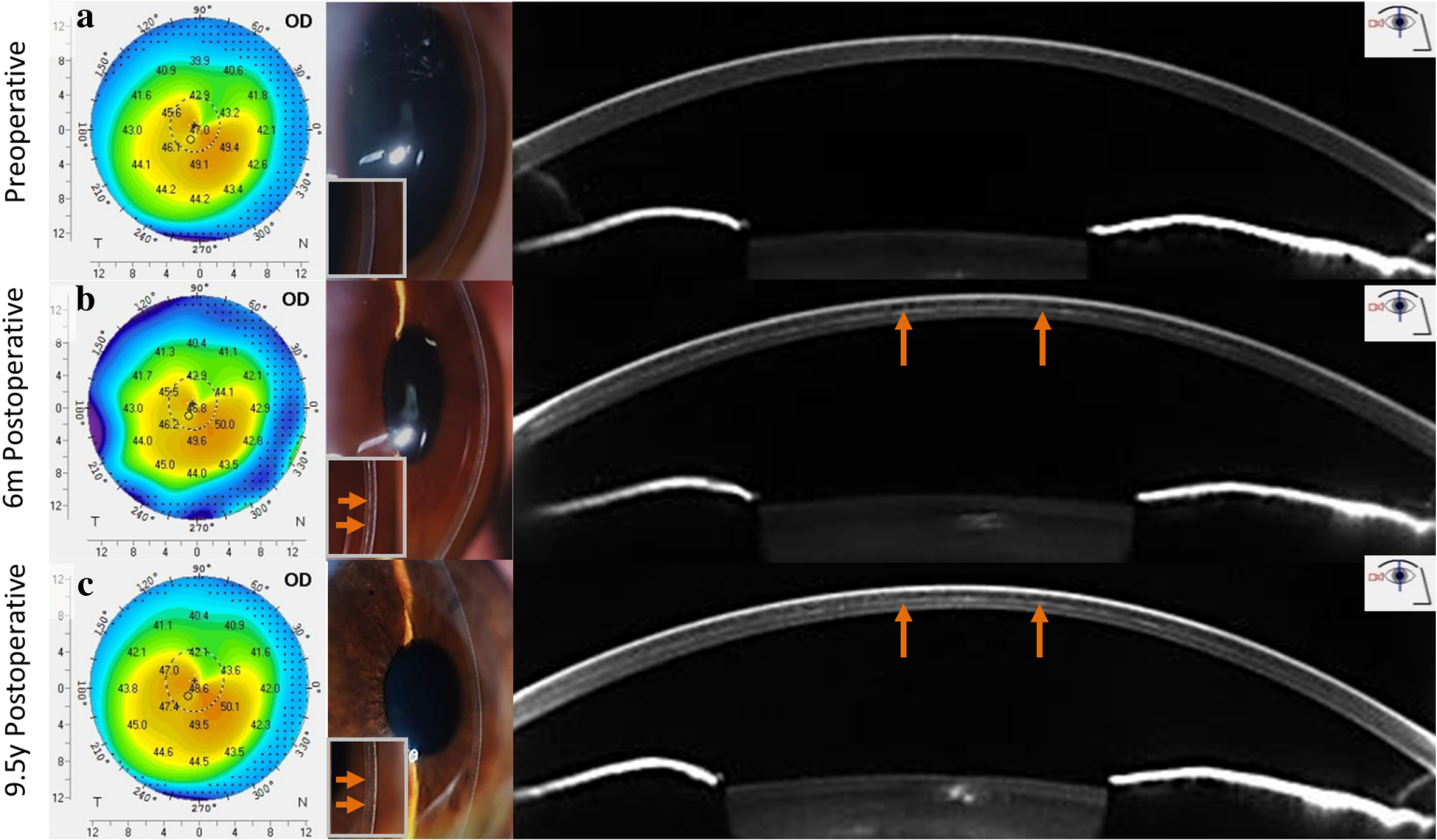
Clinical images of an eye before and up to 9.5 years after manual dissection. Topographic maps, slit-lamp images and Scheimpflug images (segment: 91°- 271°) of Case no. 1 preoperatively (top row **a**), at 6 months (second row **b**) and at 9.5 years (third row **c**) after manual mid-stromal dissection. Note a mild increase in K-readings and the demarcation line at the level of the mid-stromal dissection (arrows). m = months; y = year(s)

Manual mid-stromal dissection was derived from a technique previously described by Melles et al. to create a lamellar dissection plane in deep anterior lamellar keratoplasty (DALK) [7]. For stabilization of keratoconic corneas, a manual mid-stromal dissection plane was created at approximately 50–70% corneal depth (to avoid perforation in the anterior chamber) instead of the 90–95% depth of dissection commonly used in DALK (Fig. 1).

Surgery was performed under local anesthesia (retrobulbar, 4 mL 1% ropivacaine hydrochloride with 1 mL 150 IU Hyason) with the patient positioned in anti-Trendelenburg position and a Honan’s balloon applied for 10 min. A side port was created at either the 3- or 9-o’clock limbus to completely fill the anterior chamber with air. Then, a 5-mm frown-shaped scleral incision was created at 12 o’clock, 1–2 mm from the limbus and tunneled into the superior cornea. Subsequently, guided by the air-endothelium interface, manual lamellar dissection was performed with a dissection spatula (Melles spatula set; DORC International BV, Zuidland, The Netherlands) at 50–75% stromal depth creating a circumferential mid-stromal pocket from limbus to limbus. Finally, the air was removed from the anterior chamber and the eye was pressurized with a balanced salt solution.

Postoperative topical treatment included chloramphenicol 0.5% for 2 weeks; ketorolac tromethamine 0.4% and dexamethasone 0.1% for 4 weeks; switched to fluorometholone 0.1% at 1 month postoperatively, which was subsequently tapered and stopped over months.

### Data collection

All eyes were examined at standardized time-intervals before and after surgery: 1 day, 1 week, 1, 3, 6 and 12 months and every 6 months thereafter. Data regarding the first two postoperative years and the latest follow-up visit were included in this analysis. Slit-lamp biomicroscopy, Scheimpflug-based corneal tomography (Pentacam HR; Oculus, Wetzlar, Germany) and endothelial cell density (ECD) measurements were recorded and best-spectacle corrected visual acuity (BSCVA) and best-contact lens corrected visual acuity (BCLVA) were measured.

Regarding Scheimpflug-based corneal tomography, only images of sufficient quality were used for evaluation. BCVA was measured using a Snellen letter chart. The endothelium was photographed and evaluated in vivo using a Topcon SP3000p non-contact autofocus specular microscope (Topcon Medical Europe BV, Capelle a/d IJssel, The Netherlands). Images of the central corneal window were analyzed and manually corrected; up to three measurements of endothelial cell density were averaged (if the central endothelium could not be visualized, paracentral images were used for analysis).

### Statistical analysis

All analyses were performed using Excel Software for Windows. Progression of Kmax was defined as an increase in Kmax of ≥1.0 D throughout the follow-up period. Changes in thinnest point thickness (TPT) of less than 5% were considered stable. BCVA was defined as stable for changes ≤1 Snellen lines, and as improving or deteriorating for changes ≥2 Snellen lines. Independent paired Student’s t*-*test was performed to assess significant differences between preoperative and consecutive postoperative follow-up measurements. Statistical analysis could not be adjusted for inclusion of fellow eyes due to the small cohort size. Additional statistical analysis, excluding fellow eyes, however, yielded equal results. A *P*-value below an alpha of 0.05 was considered to be statistically significant. Reported data were expressed as mean ± standard deviation (SD) for continuous variables or percentages.

## Results

All surgical procedures were uneventful. After surgery, the mid-stromal dissection could be visualized in all treated corneas as a thin white scar by biomicroscopy (Fig. 1).

Case no. 7 was excluded from Pentacam analysis due to a preoperative measurement of insufficient quality. During the 6.6 ± 2.4 year follow-up period, 6/15 eyes (40%) showed no changes in keratometry values (simK and/ or Kmax) (Cases no. 2, 3, 4, 8, 11 and 12), while an increase of ≥1.0 D was observed in 9/15 eyes (60%) (Cases no. 1, 5, 6, 9, 10, 13, 14, 15 and 16) (Table 2). In eyes with a preoperative Kmax < 60.0 D, postoperative Kmax showed no differences compared to preoperative values in 6/11 eyes (55%), whereas an increase in Kmax > 1.0 D was observed in 4/4 eyes (100%) with a preoperative Kmax > 60.0 D. The fellow eyes of patients of whom both eyes were included were observed to behave in the same way (Cases no. 2 and 3 were both stable and cases no. 6 and 13 were both progressive). Cases no. 5 and 14 both had a preoperative Kmax of > 70.0 D and needed a subsequent Bowman layer transplantation to manage continued keratoconus progression at 47 and 19 months of follow-up, respectively, after which they were excluded from further analysis. Patient age did not correlate with disease progression (*P* ≥ .05; Table 1)*.*

**Table 2:** Pre- and postoperative corneal curvature

Case no.	Max FU years (m)	SimK-value (D)				Δ Pre-op to latest FU (D)	Kmax (D)				Δ Pre-op to latest FU (D)	Remarks
		Pre-op	1 yr. FU	2 yr. FU	Latest FU		Pre-op	1 yr. FU	2 yr. FU	Latest FU		
1	9.4 (113)	46.2	46.6	46.4	47.2	1.0	51.4	47.1	51.1	52.8	1.4^c^	
2^a^	3.1 (37)	44.8	44.5	45.1	44.5	−0.3	59.9	60.5	60.1	59.8	−0.1	
3^a^	3.0 (36)	43.5	44.5	43.2	43.3	−0.2	58.6	59.4	58.1	58.4	− 0.2	
4	9.2 (110)	45.8	45.6	n.a.	45.8	0	49.9	48.2	n.a.	50.3	0.4	
5	3.9 (47)	53.4	54.0	55.3	56.2	2.8	71.8	71.1	73.5	74.5	2.7^c^	Bowman Layer Transplantation (47 m)
6^b^	8.7 (104)	49.0	49.0	49.1	48.9	−0.1	56.5	57.1	57.8	58.5	2.0^c^	
7	8.8 (106)	*n.a.*	*(52.2)*	*(52.2)*	*(52.4)*		*n.a.*	*(58.4)*	*(57.8)*	*(58.7)*		
8	7.3 (87)	50.1	49.5	50.2	51.3	1.2	58.6	57.2	57.1	57.6	−1.0	
9	6.8 (82)	37.1	38.0	38.0	38.6	1.5	46.4	46.5	46	52.0	5.6^c^	
10	8.5 (102)	52.0	50.9	52.8	57.5	5.5	58.7	60.1	62.9	70.7	12.0^c^	
11	7.3 (87)	42.5	42.4	42.6	42.3	−0.2	53.4	52.8	53.7	52.8	−0.6	
12	8.1 (97)	47.6	46.9	n.a.	47.2	−0.4	49.4	48.3	n.a.	47.9	−1.1	
13^b^	7.7 (92)	48.3	48.1	47.8	49.2	0.9	55.5	55.9	54.6	59.9	4.4^c^	
14	1.6 (19)	61.9	60.1	n.a.	n.a.	−1.8	72.5	76.8	n.a.	n.a.	4.3^c^	Bowman Layer Transplantation (19 m)
15	6.9 (83)	58.2	57.9	60.0	61.5	3.3	69.2	70.9	78.1	74.3	5.1^c^	
16	5.8 (70)	51.1	50.8	51.2	52.2	1.1	60.3	59.9	60.1	62.3	2.0^c^	
Average	6.6 (79.5)	48.8	48.6	48.5	49.7	+ 1.0	58.1	58.1	59.4	60.6	2.5	
SD	2.4 (29.4)	6.2	5.7	6.0	6.7	1.8	7.9	9.2	8.9	9.4	3.5	
*P*-value (pre-op to FU)			0.357	0.060				0.970	0.223			

*FU* = follow-up; *Max* = maximum; *D* = diopter; *Pre-op* = preoperative; *yr*. = year; *m* = months; Δ = difference; *simK* = simulated keratometry; *Kmax* = maximum keratometry value; *SD* = standard deviation; *n.a.* = not available

^a,b^Note that two patients (cases no. 2, 3, 6, and 13) underwent bilateral manual crosslinking

^c^Progression of corneal ectasia after mid-stromal manual dissection

Case no. 7 was excluded from keratometry analysis due to a preoperative measurement of insufficient quality

No changes in central corneal thickness (CCT) or TPT were observed in 11/15 eyes (73%), whereas a decrease in TPT of more than 5% was observed in 4/15 eyes (27%) (Cases no. 9, 10, 11 and 14) (Table 3). Three of these four cases (Cases no. 9, 10 and 14) also showed an increase in keratometry values.

**Table 3:** Pre- and postoperative pachymetry values

Case no.	Max FU years (m)	Central corneal thickness (μm)				Δ Pre-op to latest FU (%)	Thinnest point thickness (μm)				Δ Pre-op to latest FU (%)	Remarks
		Pre-op	1 yr. FU	2 yr. FU	Latest FU		Pre-op	1 yr. FU	2 yr. FU	Latest FU		
1	9.4 (113)	505	502	509	490	−3.0	492	487	496	478	−2.8	
2^a^	3.1 (37)	513	510	508	509	−0.8	483	487	472	492	+ 1.9	
3^a^	3.0 (36)	534	520	530	524	−1.9	504	487	482	493	−2.2	
4	9.2 (110)	466	469	n.a.	473	+ 1.5	451	449	n.a.	444	−1.6	
5	3.9 (47)	414	410	406	404	−2.4	336	321	328	329	− 2.1	Bowman Layer Transplantation (47 m)
6^b^	8.7 (104)	470	475	473	458	−2.6	446	458	453	441	−1.1	
7	8.8 (106)	*n.a.*	*(414)*	*(419)*	*(423)*		*n.a.*	*(407)*	*(408)*	*(429)*		
8	7.3 (87)	568	571	564	557	−1.9	511	497	502	506	−1.0	
9	6.8 (82)	470	456	488	476	+ 1.3	419	344	395	379	−9.5	
10	8.5 (102)	339	350	325	329	−2.9	313	292	287	274	−12.5	
11	7.3 (87)	497	486	479	486	−2.2	481	465	456	451	−6.2	
12	8.1 (97)	471	481	n.a.	477	+ 1.3	466	480	n.a.	471	+ 1.1	
13^b^	7.7 (92)	469	467	469	457	−2.6	434	438	442	440	+ 1.4	
14	1.6 (19)	459	385	n.a.	385	−16.1	366	305	n.a.	305	−16.7	Bowman Layer Transplantation (19 m)
15	6.9 (83)	392	376	377	369	−5.9	338	332	325	322	−4.7	
16	5.8 (70)	475	453	458	453	−4.6	465	440	453	445	−4.3	
Average	6.6 (79.5)	469	461	466	456		434	419	424	418		
SD	2.4 (29.4)	56	59	67	61		65	76	73	76		
*P*-value (pre-op to FU)			0.121	0.126				0.035	0.010			

*FU* = follow-up; *Pre-op* = preoperative; *m* = months; *yr.* = year; *μm* = micrometer; *n.a.* = not available; *SD* = standard deviation

^a,b^Note that two patients (cases no. 2, 3, 6, and 13) underwent bilateral manual crosslinking

Case no. 7 was excluded from pachymetry analysis due to a preoperative measurement of insufficient quality

Pre- and postoperative BSCVA measurements were available for 12/16 eyes (75%) and remained unchanged in 7/12 eyes (58%) and improved ≥2 Snellen lines in 5/12 eyes (42%). Pre- and postoperative BCLVA measurements were available for 9/16 eyes (60%). Scleral lenses were applied in 5/9 eyes (56%), rigid gas permeable contact lenses in 2/9 eyes (22%), a soft contact lens in 1/9 eyes (11%) and one eye (11%) switched from a scleral lens to a soft contact lens. BCLVA remained stable in 4/9 eyes (44%), improved ≥2 Snellen lines in 3/9 eyes (33%) and deteriorated in 2/9 eyes (22%). The two eyes with a deterioration in BCLVA showed continued keratoconus progression and underwent subsequent Bowman layer transplantation (Cases no. 5 and 14). Mean spherical equivalent did not change from preoperatively to the latest postoperative follow-up visit (− 2.3 ± 3.8 D preoperative to − 2.4 ± 4.1 D postoperative, *P* ≥ .05). The mean refractive cylinder changed from − 3.7 ± 2.4 D to − 4.6 ± 1.2 D (*P* ≥ .05) (Table 4).

**Table 4:** Pre- and postoperative visual acuity and astigmatism

Case no.	Max FU in years (m)	BSCVA Snellen (Decimal)		BCLVA Snellen (Decimal)		Cylinder in D	SE in D	Cylinder in D	SE in D
		Pre-op	Latest FU	Pre-op	Latest FU	Pre-op	Latest FU	Pre-op	Latest FU
1	9.4 (113)	20/60 (0.3)	20/40 (0.5)	n.a.	20/40 (0.5)	−5.50	− 2.50	−6.00	−4.25
2	3.1 (37)	20/20 (1.0)	20/16 (1.2)	n.a.	n.a.	−5.00	2.50	−5.50	2.50
3	3.0 (36)	20/20 (1.0)	20/16 (1.2)	n.a.	n.a.	−5.00	2.50	−5.50	2.75
4	9.2 (110)	20/25 (0.8)	20/25 (0.8)	20/25 (0.8)	n.a.	−3.25	−7.13	−6.50	−7.50
5	3.9 (47)	20/50 (0.4)	n.a.	20/22 (0.9)	20/50 (0.4)	0.00	0.00	n.a.	n.a.
6	8.7 (104)	20/33 (0.6)	20/20 (1.0)	n.a.	20/16 (1.2)	−4.00	−0.50	−4.00	−1.25
7	8.8 (106)	20/25 (0.8)	20/25 (0.8)	n.a.	n.a.	−4.00	−2.00	−3.50	−1.50
8	7.3 (87)	20/50 (0.4)	20/28 (0.7)	20/33 (0.6)	20/25 (0.8)	−6.00	−7.00	−5.00	−2.00
9	6.8 (82)	20/28 (0.7)	20/20 (1.0)	20/25 (0.8)	20/20 (1.0)	−1.00	−5.75	−2.75	−8.50
10	8.5 (102)	n.a.	20/80 (0.25)	20/28 (0.7)	20/50 (0.4)	n.a.	n.a.	0.00	−4.00
11	7.3 (87)	n.a.	20/28 (0.7)	n.a.	20/20 (1.0)	−7.50	0.50	−6.00	0.50
12	8.1 (97)	20/20 (1.0)	20/16 (1.2)	n.a.	20/16 (1.2)	−0.50	−2.25	n.a.	n.a.
13	7.7 (92)	20/22 (0.9)	20/20 (1.0)	n.a.	20/20 (1.0)	−3.00	0.25	−3.25	−0.38
14	1.6 (19)	n.a.	n.a.	20/25 (0.8)	20/100 (0.2)	n.a.	n.a.	n.a.	n.a.
15	6.9 (83)	20/100 (0.2)	20/133 (0.15)	20/40 (0.5)	20/33 (0.6)	−6.00	−10.00	−4.25	−9.13
16	5.8 (70)	20/50 (0.4)	20/33 (0.6)	20/28 (0.7)	20/20 (1.0)	−0.50	−0.25.	−3.75	0.13

*Max* = Maximum; *FU* = follow-up; *m* = months; *BSCVA* = best spectacle corrected visual acuity; *BCLVA* = best contact lens corrected visual acuity; *D* = diopters; *SE* = spherical equivalent; *Pre-op* = preoperative; *n.a.* = not available

Endothelial cell density averaged 2670 ± 290 cells/mm^2^ preoperatively (*n* = 12) and remained stable up to the latest follow-up visit (*P* ≥ .05). No postoperative complications were observed throughout the study period.

## Discussion

In the past two decades, several surgical treatment options have been introduced aiming to delay or halt disease progression in keratoconic eyes and attempting to postpone or avoid PK or DALK [1]. UV-CXL has been shown to effectively delay progression of corneal ectasia, whereas implantation of ICRS may result in a corneal flattening, thereby improving the uncorrected visual acuity and allowing prolonged contact lens tolerance [8–13]. More recently, Bowman layer transplantation – implantation of an isolated Bowman layer into a manually dissected mid-stromal pocket – was introduced as a treatment option for corneas with advanced keratoconus (Kmax > 70 D and/or pachymetry < 400 μm) that are no longer eligible for UV-CXL or ICRS [14, 15].

The surgeries performed for this study originate from the time period preceding the technique for Bowman layer transplantation and UV-CXL approval in most countries [16, 17]. Our study suggests that it may be effective in halting corneal ectasia progression in about 50% of cases with a preoperative Kmax < 60.0 D.

Eyes ineligible for UV-CXL or ICRS due to corneal steepness and/or thickness, ocular surface disease related to atopic constitution – varying from epitheliopathy, chronic allergic conjunctivitis, cobblestone eyelids, limbal unrest – or corneal scars, may benefit from manual mid-stromal dissection as the procedure does not affect the ocular surface and does not involve a graft or synthetic implant. A further advantage of manual mid-stromal dissection may be that, apart from a thin layer of scar tissue induced, the cornea is unaltered, leaving room for all other future treatment options.

In the ophthalmic literature, the success rate of various procedures is less often stratified for different patient groups. For example, fair-skinned and blue-eyed Caucasian patients may show higher risk of epithelial wound healing problems and/or conjunctival reactibility, which comes into play with virtually all treatment options involving the ocular surface, and which determines the outcomes and incidence of postoperative complications in different geographic regions. For that reason, the choice of procedure may also depend on risk profiles for a given patient population [18].

On the other hand, our study showed that mid-stromal dissection alone fails to achieve stabilization of corneal ectasia in eyes with advanced ectasia (Kmax > 60.0 D preoperatively). For this group of eyes that was not responsive, a Bowman layer transplantation may be considered, a procedure that offers the same benefits in avoiding postoperative complications, but that does require a donor Bowman layer implant. In a recent study, 90% of eyes with progressive keratoconus and a preoperative Kmax ≥67.5 D, showed stabilization after Bowman layer transplantation [14, 19].

All the eyes included in this study, also the eyes of over 30 and even 40 years of age had documented evidence of keratoconus progression in the year preceding manual mid-stromal dissection. Progression of keratoconus beyond the age of 30 years was also confirmed in a recent study by Gokul et al. [20]. While the absence of a control group is a limitation of this study, it would be questionable and unethical to include eyes with documented progression of keratoconus without treating them, as it seems unlikely that these eyes would suddenly stabilize. A further limitation of this pilot study is the small sample size that did not allow us to analyze the clinical outcomes for different subgroups. Additional studies of larger sample size would be required to analyze the effect of manual mid-stromal dissection in different subsets of eyes.

## Conclusions

In conclusion, manual mid-stromal dissection was effective in achieving stabilization of corneal ectasia in 50% of corneas with mild to moderate progressive keratoconus. As a minimally invasive and low-risk procedure, it may, in particular, be considered in keratoconic eyes ineligible for UV-CXL or ICRS in order to postpone corneal grafting, while leaving room for all other future treatment options.

## Abbreviations

BCLVA: Best-contact lens corrected visual acuity
BCVA: Best corrected visual acuity
BSCVA: Best-spectacle corrected visual acuity
CCT: Central corneal thickness
D: Diopters
DALK: Deep anterior lamellar keratoplasty
ECD: Endothelial cell density
ICRS: Intrastromal corneal ring segments
KC: Keratoconus
Kmax: Maximum keratometry
PK: Penetrating keratoplasty
SD: Standard deviation
TPT: Thinnest point thickness
UV-CXL: Ultraviolet-A-induced collagen crosslinking
